# Healthcare-Related HCV Genotype 4d Infections in Kayseri, Turkey

**DOI:** 10.5152/tjg.2022.21822

**Published:** 2022-11-01

**Authors:** Selma Gökahmetoğlu, Ceylan Polat, Mustafa Altay Atalay, Gülten Can Sezgin, Gül Ergör, Bilgehan Aygen, Şebnem Gürsoy, Yusuf Hakan Abacıoğlu

**Affiliations:** 1 Department of Medical Microbiology, Erciyes University Faculty of Medicine, Kayseri, Turkey; 2 Department of Medical Microbiology, Hacettepe University Faculty of Medicine, Ankara, Turkey; 3 Department of Gastroenterology, Erciyes University Faculty of Medicine, Kayseri, Turkey; 4 Department of Public Health, Dokuz Eylül University Faculty of Medicine, İzmir, Izmir, Turkey; 5 Department of Infectious Diseases, Erciyes University Faculty of Medicine, Kayseri, Turkey; 6 Department of Medical Microbiology, İzmir University of Economics Faculty of Medicine, İzmir, Turkey

**Keywords:** HCV, genotype 4, molecular epidemiology, risk factor, Turkey

## Abstract

**Background:**

The frequency of genotype 4 hepatitis C virus infection is significantly higher in a city compared to other provinces in Turkey. In this study, we aimed to investigate the epidemiology and risk factors of hepatitis C virus genotype 4 infection in Kayseri province of Turkey.

**Methods:**

A case–control study was conducted with 61 hepatitis C virus genotype 4-infected patients and 71 controls. A questionnaire was administered to the patients and controls, asking for information about the risk factors of hepatitis C virus transmission. Core/E1 and NS5B regions of hepatitis C virus genome were amplified and sequenced by Sanger method. Phylogenetic analysis and molecular clock analysis were performed. The risk was determined by calculating the odds ratio and 95% CI. Logistic regression analysis was performed to determine the effect of risk factors by controlling for confounding variables.

**Results:**

Kayseri isolates were closely related to type 4d sequences but formed a separate cluster. According to the molecular clock analysis, hepatitis C virus genotype 4d entered Kayseri province probably between 1941 and 1988. Blood transfusion and surgical intervention were found to be significant risk factors for the infection.

**Conclusion:**

Epidemiological data showed that hepatitis C virus genotype 4d infections are significantly associated with unsafe medical procedures.

**Main Points**

Hepatitis C virus (HCV) type 4d infections could be related to unsafe medical procedures unlike in Europe.
HCV genotype 4d entered Kayseri province probably between 1941 and 1988.
Phylogenetic data of HCV genotype 4d were similar with the previous studies done in Kayseri.

## Introduction

Hepatitis C virus (HCV) can cause both acute and chronic hepatitis, ranging in severity from a mild illness to serious illness, including liver cirrhosis and cancer. Globally, an estimated 58 million people have chronic hepatitis C virus infection, with about 1.5 million new infections occurring per year. Hepatitis C virus is a blood-borne virus and most infections occur through exposure to blood from unsafe injection practices, unsafe health care, unscreened blood transfusions, injection drug use, and sexual practices that lead to exposure to blood.^1^

There are 8 genotypes of HCV and 86 subtypes based on genomic sequence heterogeneity.^2,3^ Genotypes 1, 2, and 3 have a worldwide distribution and account for most HCV infections in Europe, North America, and Asia. Genotype 4 is most common in the Middle East and North and Central Africa. Genotype 5 is found primarily in South Africa and genotype 6 occurs throughout Asia. Genotypes 5, 7, and 8 originate in southern and central sub-Saharan Africa.^1–3^ Therefore, genotyping before the treatment has become a standard of care for patient management.

Sanger sequencing is considered a gold standard for HCV genotype and subtype determination. Methods based on 5’UTR sequences are generally acceptable for genotype determination but should be carefully designed for subtyping due to the degree of sequence conservation between different viruses. Further virtue subtype identification can be achieved through analysis of the NS5B, core, and/or core-E1 genes.^1^

Hepatitis C virus genotype 1 and especially type 1b virus causes approximately 90% of these infections in Turkey, while types 2, 3, and 4 exist, still in low prevalence.^4^ It has been shown that genotype 1b infections in Turkey are associated with healthcare services.^5^ Barut et al^6^ investigated the risk factors of HCV infection; most of the patients were female and previous hospitalization was a more common risk factor in female cases compared to males. Recent studies have shown that HCV epidemiology has changed in Turkey and genotype 3 infections have occurred due to intravenous drug use.^7,8^

There are 2 reports from Kayseri, where is a relatively large province in Central Anatolia Region, that indicated unusual high prevalence of genotype 4 infections, reaching 35% among patients applied to the hospital for treatment of chronic hepatitis C.^9,10^

In Europe, most of the genotype 4d patients were male and intravenous drug users.^11,12^ It was shown that Kayseri type 4 isolates were closely related to subtype 4d sequences, however, epidemiology and risk factors of HCV genotype 4d infection are not known. In this study, it was aimed to investigate the epidemiology and risk factors of HCV genotype 4 infection in Kayseri province of Turkey.

## Materials and Methods

### Study Population and Sample Collection

A case–control study was designed to investigate the risk factors for HCV. Sixty-one treatment-naive and genotype 4-infected chronic hepatitis C patients, who were admitted to Erciyes University, Departments of Gastroenterology and Infectious Diseases in 2010-2016, were included in the study.

There were 71 controls, matched to the patients for age within 8 years, gender who were selected among people living in the same neighborhood as the patients. All of the controls were anti-HCV negative. A questionnaire was administered to the patients and controls which contained information regarding the socioeconomic characteristics and risk factors of HCV transmission routes.

This study was approved by Erciyes University Ethics Committee of Clinical Research (number: 2012/411). Informed consent was obtained from all patients.

### Molecular Analysis

Extraction of viral RNA from serum samples was performed with the EZ-1 Virus Mini kit V.2.0 (Qiagen, Germany) on the EZ-1 platform. Ten microliters of RNA extract was reverse-transcribed using random hexamers with the High-Capacity cDNA Reverse Transcription Kit (ABI Prism, Applied Biosystems, Foster City, CA, USA) according to the manufacturer’s recommendations.

Nested polymerase chain reaction (PCR) was applied to amplify the 472 bp in the Core/E1 gene between positions 843 and 1315 (GenBank accession number AF 009606) using primers 108, 109, 110, and 111.^13^ The primers 110 and 111 were used in the first PCR, the primers 108 and 109 were used in the second PCR. A heminested PCR was applied to amplify the 380 bp stretch in the NS5B gene between positions 8256 and 8636 (GenBank accession number AF 009606) using primers PR3.1, PR4, and PR5.^14-16^

Polymerase chain reactions were carried out in volumes of 20 µL using 10 µL Amplitaq Gold Fast (ABI Prism, Applied Biosystems), 10 pmol sense and antisense primers, 2 µL cDNA for the first PCR, and 3 µL PCR product for the second PCR. The cycling parameters for amplification of Core/E1 gene^13^ and for NS5B gene^16^ were applied as indicated in the literature.

Polymerase chain reaction products were visualized in 1% agarose gels stained with ethidium bromide and then purified using a PCR purification kit (Invitrogen, Waltham, MA, USA). The sequencing was done using Big Dye Sequencing Chemistry with primers; PR3.1, PR5, 108, and 109. ABI sequencer 3130 (ABI Prism, Applied Biosystems) was used to generate the sequences. The sequence analysis was performed bidirectionally.

Phylogenetic analysis was done with the sequences of Kayseri isolates and reference sequences from different genotypes downloaded from the Los Alamos HCV database.^17^ All sequences were aligned using the Clustal W modality provided in MEGA 5.02 software.^18^ Phylogenetic trees were established using gamma distributed with invariant sites parameter, general time-reversible model, and maximum likelihood methods in MEGA 5.02 software.^18^ Bootstrap analysis (1000 replicates) was applied to check the reliability of the trees.

### Molecular Clock Analysis

MEGA software 5.02 was used to determine molecular clock analysis.^18^ Evolutionary rates reported in the literature for the Core/E1 region and NS5B region were used.^19,20^ The maximum likelihood method was applied to estimate the date of entry of HCV genotype 4 into Kayseri province, Turkey.

### Statistical Analysis

In the data analysis, the variables specified by count were expressed in terms of number, percentage, and the variables determined by measurement were expressed as mean ± standard deviation. The chi-square test was used for categorical variables when evaluating the association of independent variables with the dependent variable. The dimension of risk was determined by calculating the odds ratio (OR) and 95% CI. Logistic regression analysis was performed to determine the effect of risk factors by controlling for confounding variables. Logistic models included variables that were significant in literature and in univariate analysis. Hosmer–Lemeshow test was used for model fit. In all the analyses, *P *< .05 was considered statistically significant. Data were evaluated with the Statistical Package for Social Sciences version 20.0 software (IBM Corp.; Armonk, NY, USA).^21^

## Results

### Analysis of Risk Factors Affecting HCV Genotype 4 Infection

All of the patients were citizens of Kayseri province and 85.2% of them were residing in the city center. The mean ages of patients were 57.08 ± 12.84 years; mean levels of HCV RNA viral load were 6.28 ± 1.22 log 10 IU/mL. Mean levels of ALT and AST were 55.61 ± 38.33 and 67.04 ± 58.45, respectively. Histologic activity index and fibrosis results were 5.54 ± 3.4 and 1.66 ± 1.6, respectively.

Univariate analysis of risk factors affecting HCV genotype 4 infection is shown in Table 1. None of the patients and controls were intravenous drug users and had hemodialysis treatment and got a tattoo. None of the patients had acupuncture and manicure–pedicure. None of the controls had sexually transmitted diseases. Risk factors found to be statistically associated with HCV genotype 4 infection were having a history of hospitalization, biopsy and endoscopy, blood transfusion, surgery, injection, sexual intercourse using a condom, primary school or lower education, and being a housewife and worker (*P *< .05, Table 1). Surgical interventions were applied to patients before 1996. In addition, patients with a history of blood transfusion were transfused before 1996. Multivariate analysis of factors affecting HCV genotype 4 infection is shown in Table 2.

When it is controlled for education and gender, blood transfusion (odds ratio (OR) = 10.9, 95% CI 3.17, 3.26) and surgical intervention (OR = 4.03 95% CI 1.64, 9.92) increases the risk of HCV genotype 4 infection significantly (Table 2).

### Phylogenetic Analysis

**Figure 1.**
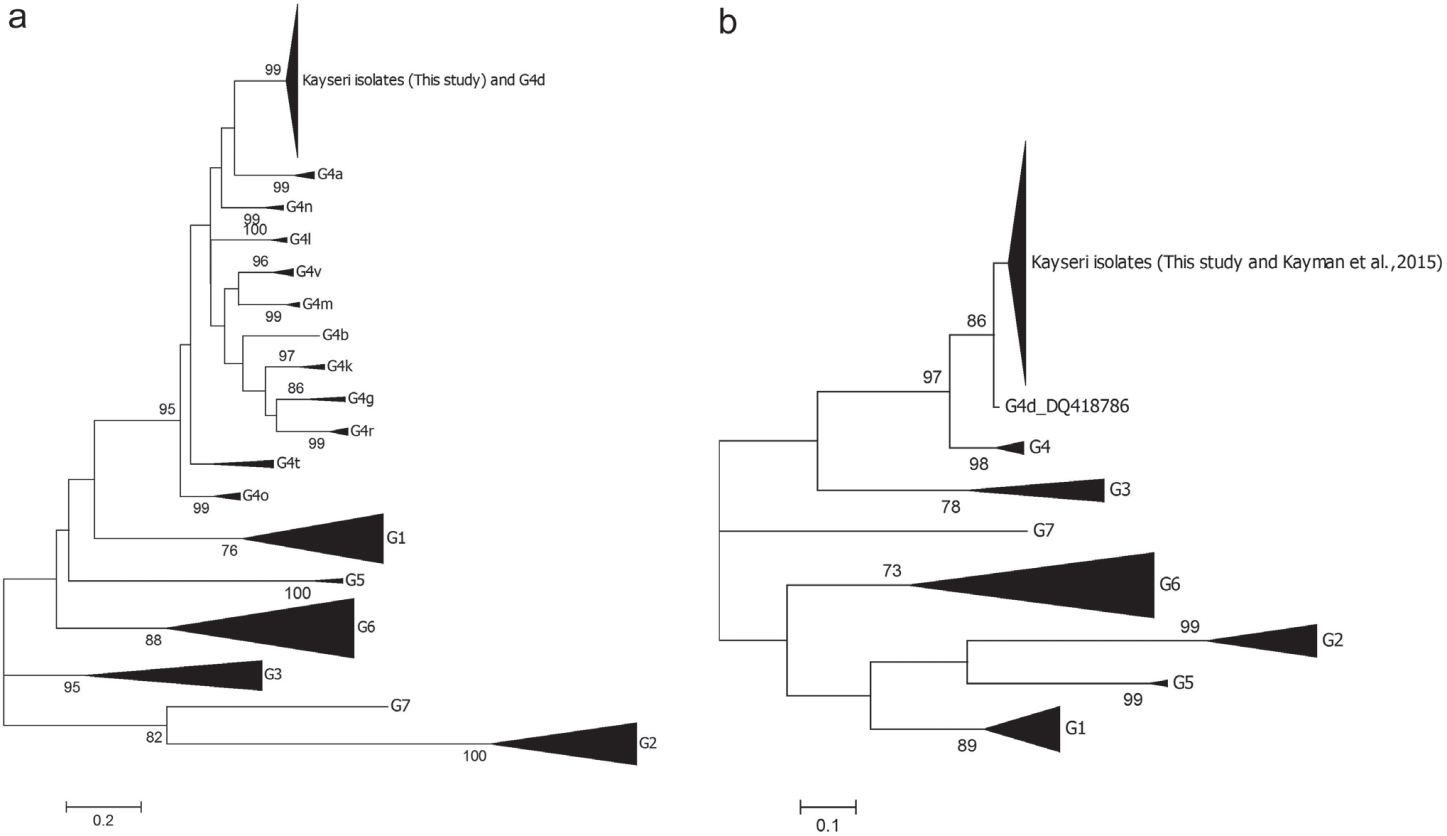
(a) The phylogenetic trees of Core/E1 nucleotide sequences from HCV genotype 4d Kayseri isolates in this study. (b) The phylogenetic trees of NS5B nucleotide sequences from HCV genotype 4d Kayseri isolates in this study and Kayman et al^16^ 2015.” The trees were constructed using Maximum Likelihood method with the General Time Reversible (GTR) model, Gamma distributed with Invariant sites (G+I) for 1000 replications. Bootstrap values lower than 70 are hidden.

Sixty Core/E1 and 50 NS5B sequences were obtained from the 61 samples. The other sequences were not clear enough for the phylogenetic analyses. Hepatitis C virus genotype 4 isolates were closely related with type 4d sequences but formed a distinct cluster (Figure 1a and b). All the sequences found in this study were deposited into GenBank. They were assigned the accession numbers MH899456 to MH899505 for NS5B and MH899506 to MH899565 for Core/E1 sequences.

At the nucleotide level, Kayseri isolates differ from each other by 5.4% for Core/E1 and 4.1% for NS5B. The distances of the isolates from the HCV genotype 4d isolates were 9.2% and 6.6% for Core/E1 and NS5B, respectively. The NS5B sequences of Kayseri isolates were 4.2% distant from the isolates which were reported in Kayman et al^16^ based on the analysis of identical regions.

According to the molecular clock analysis, HCV genotype 4d entered Kayseri province probably between 1941 and 1988.

## Discussion

Hepatitis C virus genotype 1, and specifically 1b, accounts for most HCV infections in Turkey. Genotypes 2, 3, and 4 infections are found at a low rate.^4^ Kayseri province has an unique HCV genotype distribution, genotype 4 infections account for at least 1/3 of HCV infections.^9,10^ Recent analysis showed that type 4 sequences belong to 4d subtype.^9^ In that study, 68% of HCV genotype 4d patients were over the age of 40 and female; the risk factors of HCV genotype 4d infection were not determined. We did not know how the HCV genotype 4d infection spreads, these infections may be healthcare related. It has been shown that genotype 1b infections in Turkey are associated with healthcare services.^5^ Barut et al^6^ investigated the risk factors of HCV infection; most of the patients were female and previous hospitalization was a more common risk factor in female cases compared to males. In our study, it was aimed to investigate the epidemiology and risk factors of HCV genotype 4d infection. According to the results of this study, 60% of patients were female and 97.3% of female patients had birth/abortion in hospitals. Unsafe medical procedures in healthcare services can be the cause of the predominance of female patients. Also, the risk factors found to be statistically associated with HCV 4 infection were having a history of hospitalization, biopsy and endoscopy, blood transfusion, surgery, injection, sexual intercourse without using a condom, primary school or lower education, and being a housewife and worker (*P *< .05, Table 1). However, the hospitalization and biopsy in patients with HCV 4d can be due to reverse causality that patients had a biopsy because of HCV infection and are more likely to be hospitalized. Since the patients were HCV positive, it is thought that they had intercourse using condoms more than the control group. In multivariate analysis, blood transfusion and surgical intervention were found to be significant independent risk factors for HCV genotype 4d infection. Another study from Turkey reported that surgical intervention, blood transfusion, multiple sexual partners, and dental treatment were more frequent in patients with chronic HCV infection compared to the control group.^22^ However, the genotypes were not reported in that study.

A study from Mexico reported that intravenous drug use, blood transfusion, and tattooing are important risk factors when compared to patients with HCV infection and controls.^23^ In our study, there were no drug users and tattoo history in any of the patients or controls. This indicates that the risk factors influencing HCV transmission vary from country to country according to the geographical area. Murphy et al^24^ did a case–control study in the US that identified risk factors for HCV infection in blood donors. Injecting drug use, blood transfusion among non-injecting drug users, and sexual act with injecting drug users were found to be risk factors for HCV infection.^24^

In Turkey, HCV antibody screening of blood donors was begun in 1996. Anti-HCV positivity in blood donors from various centers was found to be 0.6% between 1991 and 1996; recently anti-HCV positivity among blood donors was found to be 0.07%.^25^ The reduction in HCV prevalence has been attributed to a higher public awareness than in previous years as a result of intensive education and campaigns. Professional and public health education and implementation of infection control practices in all health facilities are of utmost importance.

Kayman et al^16^ detected that the subtype of genotype 4 isolates as 4d in Kayseri. In this study, we investigated the molecular epidemiology of HCV genotype 4 isolates. The isolates were type 4d and formed a separate cluster. Phylogenetic data of this study were similar to the study of Kayman et al^16^ based on the analysis of identical regions (Figure 1b).

In Europe, most of the genotype 4d patients were male and intravenous drug users.^11,12^ Unlike in Europe, patients and controls had no history of intravenous drug use and most genotype 4d patients were older women in our study like Kayman’s study.^9^ In Turkey, the most common HCV genotype is type 1b and HCV infections are more common in older women due to unsafe medical procedures.^5,6^ Recent studies have shown that HCV epidemiology has changed, genotype 2 and 3 infections have increased and especially genotype 3 infections, which are seen in young men on contrary to our work and previous studies, have occurred due to intravenous drug use in Turkey.^7,8^

Cantaloube et al^26^ showed that genotype 4d infections grow exponentially from the 1960s to 1980. However, Ciccozzi et al^27^ reported that the genotype 4d epidemic in Southern Italy had been maintained in a steady non-expanding phase until the late 1970s after that it grew exponentially up to the 1990s. HCV genotype 4d isolates from Kayseri province were analyzed together with European type 4d isolates and Kayseri isolates had a different origin being completely separated from the European one.^15^ In the same study, the demographic history of Turkish HCV 4d isolates showed that the epidemic started in 1970s, then following a slow exponential growth until 1980s and after that the plot reached a plateau which still remains today. According to molecular clock analysis, HCV genotype 4d entered Kayseri province between 1941 and 1988, similar to Ciccozzi et al^15^. This period coincides with the significant increase in the number of medical procedures, such as blood transfusions and unsafe therapeutic injections, which are the main cause of the worldwide spread of HCV.^28,29^ The plateau reached by the epidemic in the early 1990s seems to indicate the partial success of the new transfusion policies adopted in Turkey.

Cicozzi et al^15^ showed that HCV 4d gene flow mainly occurred from Kayseri to the periphery according to the migration pattern analysis.^15^ The risk factors of HCV genotype 4d infection were not identified in that study, but the risk factors were determined in our study and the data on healthcare-related infection suggests that the infection is from the center to the periphery. It is known that a lot of patients from the periphery of Kayseri province come to the center of the city to get health services.

Our study is the first case control study that investigates the risk factors of HCV genotype 4d infection. The risk factors strongly suggest that genotype 4d infection is healthcare related. As HCV genotype 4d entered Kayseri between 1941 and 1988, patients most probably contracted the virus in those years in a common healthcare facility. Since the exposure was many years ago, it is not easy to get specific and reliable information from patients. However, we still had statistically significant results for some of the risk factors. In conclusion, epidemiological data showed us that HCV type 4d infections could be related to unsafe medical procedures and phylogenetic data were similar to the previous studies done in Kayseri.

## Figures and Tables

**Table 1.** Univariate Analysis of Risk Factors Affecting HCV Genotype 4 Infection

	**Patient (%)**	**Control (%)**	**OR**	**GA**	***P***
**Gender**					
Female	37 (60.7)	44 (62)	1.06	0.52-2.13	.877
Male	24 (39.3)	27 (38)			
**Place of residence**					
Center	52 (85.2)	62 (87.3)	1.19	0.44-3.22	.729
Out of center	9 (14.8)	9 (12.7)			
Married	55 (90.2)	67 (94.4)	1.83	0.49-6.80	.512
History of travel					
Living abroad	10 (16.4)	9 (12.7)	1.35	0.51-3.58	.545
Travelling abroad	20 (32.8)	17 (23.9)	1.55	0.72-3.33	.261
Living abroad of partner	11 (20)	8 (11.9)	1.84	0.68-4.97	.226
Travelling abroad of partner	15 (27.3)	15 (22.4)	1.30	0.57-2.97	.534
History of hospitalization	50 (82)	40 (56.3)	3.52	1.577-7.869	.002
Biopsy, endoscopy	54 (88.5)	19 (26.8)	21.11	8.193-54.404	<.001
**Risk factors**					
Dormitory stay	7 (11.5)	3 (4.2)	2.94	0.73-11.90	.131
Blood transfusion	24 (39.3)	4 (5.6)	10.87	3.50-33.70	<.001
Dental treatment	57 (93.4)	63 (88.7)	1.81	0.52-6.33	.353
Surgical intervention	46 (75.4)	32 (45.1)	3.74	1.77-7.89	.001
Birth/abortion (patient n = 37; control n = 44)	35 (97.3)	42 (95.5)	1.71	0.15-19.70	.665
Shared razor	13 (21.3)	7 (9.9)	2.48	0.92-6.68	.073
Shaving in the barber (patient n = 24; control n = 27)	17 (70.8)	21 (77.8)	1.44	0.41-5.10	.57
Injection	23 (37.7)	5 (7.0)	7.99	2.81-22.75	<.001
Intercourse using a condom	24 (39.3)	4 (4.2)	10.7	3.64-38.39	<.001
Number of sexual partners (more than 1 partner)	5 (8.2)	2 (2.8)	3.08	0.58-16.48	.189
**Age (years)**					
≤39	5 (8.1)	7 (9.9)	Ref	-	-
40-54	13 (21.3)	16 (22.5)	1.12	0.32-4.80	.92
55-70	32 (52.4)	41 (57.7)	1.13	0.30-4.11	.92
≥71	11 (18.0)	7 (9.9)	2.13	0.54-10.32	.31
**Education**					
Primary school or lower	45 (73.8)	38 (53.5)	2.80	1.10-7.51	.03
High school	8 (13.1)	14 (19.7)	1.42	0.42-4.60	.60
University	8 (13.1)	19 (26.8)	Ref	-	-
**Occupation**					
White collar	3 (4.9)	5 (7.0)	1.92	0.2-16.5	.80
Housewife	36 (59.0)	33 (46.5)	3.54	1.1-13.5	.04
Worker	15 (24.6)	7 (9.9)	6.63	1.6-31.4	.01
Professional	4 (6.6)	13 (18.3)	Ref	-	-
Others	3 (4.9)	13 (18.3)	0.80	0.1-4.0	>.99

**Table 2.** Multivariate Analysis of Factors Affecting HCV Genotype 4 Infection

	**B**	**OR**	**95% CI**	
			**Lower**	**Upper**
Education	0.015	-	-	-
Education 1^*^	1.921	6.82	1.79	26.03
Education 2^**^	0.834	2.30	0.55	9.69
Gender	0.282	1.32	0.51	3.48
Blood transfusion	2.386	10.86	3.17	37.26
Surgical intervention	1.395	4.03	1.64	9.92

^*^Primary school or lower.

^**^University.

HCV, hepatitis C virus.
